# MEDICOLEGAL ARTICLE

**Published:** 2010

**Authors:** S. C. Parakh

**Affiliations:** Hyderabad, India E-mail: scparakh@yahoo.co.in

**Are we taking a legally valid “Informed Consent”?**

All anaesthesiologists ensure that consent form has been signed by the patient before administering anaesthesia; however, very few attempt to know whether the consent they are getting is legally valid or not.

The word *informed* implies that the patient has been communicated about the general and specific risks involved in anaesthesia and surgery. Risks specific to the patient are because of his or her surgical problem, coexisting medical problems and other factors and will vary from patient to patient. Therefore, there cannot be a pre-printed universal consent form (unless details of all possible techniques, medical problems and their possible complications are incorporated in it).

Moreover, information should be given to the patient in a language and form that he can understand. India being a multilingual nation, the consent form should mention the language in which the information was given to the patient.

Therefore, a consent form could be partly printed, with certain information to be filled by hand in the blank spaces. It should include the following:

1. Particulars of the patient (name, age, sex, address, etc.)
2. Surgical procedure planned
3. Anaesthetic technique planned (alternative techniques and reasons for selecting a particular technique)
4. Name of surgeon and anaesthesiologist if any assurance has been given that a particular surgeon or anaesthesiologist will be involved
5. Anticipated risks (general and specific to the patient due to his or her personal factors)
6. Acceptance or refusal by the patient
7. Language in which the information is given
8. Name of person who gave the information and obtained the consent

At the bottom, following persons should sign:

1. The patient (parent or guardian or next of kin if patient is not legally competent to sign the consent)
2. Attendant of the patient (as a witness)
3. Person who is obtaining the consent (hospital staff)

